# Is Cytoreductive Nephrectomy Still a Standard of Care in Metastatic Renal Cell Carcinoma?

**DOI:** 10.15586/jkcvhl.2019.114

**Published:** 2019-03-05

**Authors:** Alex Renner, Suraj Samtani, Arnaldo Marín, Mauricio Burotto

**Affiliations:** 1 Medical Oncology Department, University of Chile Clinical Hospital, Santiago, Chile; 2 Oncology Unit, Clinica Alemana, Santiago, Chile; 3 Basic & Clinical Science Department, University of Chile, Santiago, Chile; 4 Oncology Department, Los Andes University, Bradford Hill Clinical Research Center, Santiago, Chile

**Keywords:** cytoreductive nephrectomy, immunotherapy, metastatic renal cell carcinoma, sunitinib, surgical outlook

## Abstract

Cytoreductive nephrectomy has been an integral part of management in metastatic renal cell carcinoma for patients with good performance status, based on the benefit shown by prospective trials in the interferon era and retrospective trials in the targeted therapies era. Clinical Trial to Assess the Importance of Nephrectomy (CARMENA), the first prospective phase III trial comparing a targeted agent alone (sunitinib) versus nephrectomy plus sunitinib, has been recently published, showing non-inferiority for the nephrectomy-sparing arm. In this article, we discuss the impact of nephrectomy including its immune-mediated effects, surgical morbidity and mortality, and the clinical data supporting the indications of nephrectomy in order to analyze the CARMENA trial in context, with the aim to identify optimal strategies for different patient populations in the metastatic setting.

## Introduction

Kidney cancer is the ninth most common cancer in men and the 14th in women, with an estimated 338,000 new cases worldwide each year; it is also the 16th most common cause of death from cancer, causing 143,000 deaths in 2012 (1). Renal cell carcinoma (RCC) accounts for approximately 90% of all kidney malignancies; the most common histological type being clear cell carcinoma, which is present in 80–90% of cases. Median age at diagnosis is 64 years, and 5-year survival rates have been steadily increasing over time, from 50.1% in 1977 to 74.4% in 2014, mainly reflecting stage migration to earlier tumors diagnosed incidentally through image tests. This 5-year survival goes down to just 11.6% for metastatic disease according to the US Surveillance Epidemiology and End Results (SEER) database (2).

The only known curative therapy for RCC localized within the kidney is complete surgical resection of the tumor, via radical or partial nephrectomy. At diagnosis, up to 30% of patients present with metastatic renal cell carcinoma (mRCC) (3). In the metastatic setting, cytoreductive nephrectomy (CN) has been a standard of care for fit patients since randomized controlled trials in the interferon era (4, 5) showed an overall survival (OS) benefit for patients undergoing nephrectomy followed by systemic treatment versus systemic therapy alone. However, systemic therapy for mRCC has changed significantly in the last 15 years, with several new active agents available. Thus, the role and timing of nephrectomy has been questioned, given the surgical risks and potential delays in systemic treatment (6). The evidence for CN in the targeted therapy era was, until recently, based purely on retrospective studies which have suggested that the OS benefit still exists.

Since new data from prospective SURTIME and CARMENA trials are available, we believe it is an appropriate time to reassess this subject and try to define which patients may or may not benefit from CN in the mRCC setting with currently available therapies.

## Biological Effects of Nephrectomy

Even though CN proved beneficial in prospective trials in the interferon era, the mechanism for the observed benefit is still not completely understood. Several studies have pointed to the immunologic dysfunction present in mRCC, which could be mitigated by removal of the primary tumor.

Lahn et al. (7) describe the significant elevation of circulating proinflammatory and T-cell inhibitory cytokines such as interleukins 6, 8 and 10 and TNF-α. Uzzo (8) indicates that FasL expression by the tumor may be responsible for the increased T-cell apoptosis seen in these patients. Natural killer cell dysfunction mediated by regulation of the TGF-β/SMAD pathway to evade innate immune surveillance has been recently described (9). Dadian (10) measured peripheral immunological parameters pre- and post-nephrectomy, showing a decreased inflammatory response, improved natural killer activity and increased immune activation after surgery. Also, inflammation and immune evasion mediated by elevated secretion of CCL1 by the tumor and increased presence of CCR8 (+) myeloid cells in peripheral blood and cancer tissues have been shown by Eruslanov (11). Ongoing trials such as NCT02446860 (12) are measuring changes in immune markers before and after neoadjuvant PD-1 blockade followed by nephrectomy and are trying to correlate them to both response and toxicity.

Besides the potential immunologically mediated effects of nephrectomy, Gatenby (13) proposed that metabolic acidosis associated with mild renal failure after unilateral nephrectomy can decrease tumor growth and invasion. Regarding the impact of nephrectomy on response rates to systemic treatment, the Flanigan et al. trial (4) which compared interferon plus CN versus CN alone showed that response rates to interferon remained at a very low 3.3 and 3.6% respectively. On the first-line nivolumab plus ipilimumab versus sunitinib trial (14), 660 out of 859 patients (76.8%) underwent CN; here, the subgroup analysis shows that the Hazard Ratio (HR) for death was 0.69 (0.53–0.89) in those who underwent nephrectomy and 0.63 (0.42–0.94) in those with no nephrectomy, showing the benefit from immunotherapy does not appear to differ between these subgroups.

## Surgical Outlook

Nephrectomy, as with most oncologic surgical procedures, aims to achieve removal of gross tumor burden to increase survival and reduce symptoms. Before the era of early immunotherapy, nephrectomy in the mRCC setting was mostly reserved for patients with only one metastatic lesion, and to control cancer-related symptoms such as hematuria, flank pain, and paraneoplastic syndrome (15, 16). As we moved into the interferon era, the use of CN increased significantly, owing to the improved OS shown in prospective trials. In a SEER registry analysis, Conti et al. (17) reported that the proportion of patients undergoing CN in the metastatic setting increased from 29% in 1993 to 39% in 2004; after that year (and coinciding with the introduction of targeted therapies), a slight but progressive decrease was observed, less than 34% by 2010.

Regarding complications, CN can be a challenging procedure, with potential morbidity and mortality. Silberstein et al. (18) analyzed 195 mRCC patients who underwent nephrectomy at Memorial Sloan-Kettering Cancer Center (MSKCC) between 1989 and 2009, reporting that 27% of them developed grade ≥ 2 and 8% grade ≥ 3 complications within 8 weeks of surgery; those who had complications were significantly less likely to receive systemic therapy within 56 days (OR 0.32; P = 0.024), and the best predictors for postoperative complications were Karnofsky Performance Scale (KPS) and age, even more so than other performance measures like MSKCC risk categories. Abdollah et al. (19) reviewed results on 1063 patients undergoing CN and found that in-hospital mortality was 2.4% and complications were present in 26.5% of cases. Wallis et al. (20) reported a 3.2% mortality rate in a prospective registry in the USA. Using the SEER database, Cloutier et al. (21) demonstrated a 30-day mortality of 4.2% in mRCC patients (all T stages) compared with just 0.3% for T1-2N0M0 and 1.3% for T3-4N0-2M0 disease; mortality reached 10.5% in the subset of patients aged 80 years or older. Similarly, Sun et al. (22) confirmed that elderly patients (>75 years) were 2.2-fold more likely to experience perioperative mortality than younger patients. You et al. (23) showed that in patients with two or more risk factors (KPS < 80, hemoglobin less than the lower limit of normal, neutrophils greater than the upper limit of normal, and clinical N2 stage), overall survival was not modified by CN. As we can see, patient selection is critical in order to differentiate patients who may benefit from those who may not.

## Evidence for Cytoreductive Nephrectomy in the Interferon Era

With the emergence of interferon (IFN) and interleukin-2 (IL-2) immunotherapy, the role of CN was reevaluated in two prospective randomized trials. The SWOG 8949 trial by Flanigan et al. (4) evaluated overall survival in 241 treatment-naïve patients, who were randomized to either nephrectomy followed by IFN or IFN alone; the groups were imbalanced, with more ECOG PS 0 patients in the surgery arm, which by itself may have accounted for their better survival. Median overall survival (mOS) was 11.1 versus 8.1 months favoring the surgery plus interferon arm, with overlapping confidence intervals and a borderline P-value (P = 0.05). Another prospective trial was EORTC 30947 by Mickisch et al. (5) which compared 42 patients undergoing surgery plus IFN versus 43 patients who received IFN alone, showing an OS of 17 versus 7 months, respectively (P = 0.03, HR 0.54, 95% CI 0.31–0.94).

Since EORTC 30947 and SWOG 8949 had identical protocols, a combined analysis was later published (6) including all 324 patients. The PS imbalance remained (PS 0 = 53.4% in the surgery + IFN arm versus 41.1% in the IFN alone arm), otherwise both arms were well balanced. Twelve percent of patients randomized to nephrectomy and IFN did not undergo surgery and another 5.6% in this arm did not receive IFN, but they were all included in the intention to treat analysis. Median OS was 13.6 months for nephrectomy plus IFN versus 7.8 months for interferon alone (HR 0.69, 95% CI 0.55–0.87, P = 0.002). The OS benefit was present in both PS 0 and 1 patients, but more pronounced for those in PS 0. Objective response rates (ORR) were low and did not differ between both arms: 6.9% in the nephrectomy plus IFN group versus 5.7% in the IFN alone group (P = 0.60). Surgical complications were present in 23.4% of patients that underwent nephrectomy, including two surgical deaths (1.4%).

Once these data became available, nephrectomy became standard of care for patients with mRCC and PS 0-1. This was further supported by retrospective analyses showing nephrectomy as one of the several prognostic factors associated with improved OS. Motzer (24) performed a retrospective study on 670 patients with advanced RCC treated in 24 successive clinical trials during 21 years at the MSKCC. Out of the six parameters identified as risk factors in the multivariate analysis, “absence of nephrectomy” had the least powerful association, but it was still significant, with a risk ratio of 1.35 for death (CI 95%, 1.13–1.61, P = 0.01).

## Evidence for Cytoreductive Nephrectomy in the Targeted Therapies Era: Retrospective Data

In the last 15 years, there has been an explosive growth in the available systemic therapies for the management of mRCC, owing to the better understanding of molecular mechanisms involved in the development of renal cancer. Several agents targeting the vascular endothelial growth factor (VEGF) such as sunitinib (3), pazopanib (25), and bevacizumab (26) have proven to be effective in randomized trials. Mammalian target of rapamycin (mTOR) inhibitor temsirolimus has also shown superiority over IFN in patients with poor prognosis (27). More recently, drugs targeting the immune checkpoint inhibitor programmed death 1 receptor (PD-1) and its ligand (PDL-1) have also shown significant activity in RCC, alone or in combination with other agents (14). In pivotal trials for all these novel drugs, CN was performed in over 80% of patients, thus no analysis on the effect of surgery can be made based on their results.

Since effective therapy became available, delaying treatment to perform a nephrectomy has become a concern. Several large retrospective studies have reviewed the effect of nephrectomy for patients treated with modern targeted therapies, which are summarized in Table 1. Hanna et al. (28) evaluated data from the USA National Cancer Database between 2006 and 2013 in which nephrectomy was performed in 35% of mRCC patients analyzed (5374 out of 15,390). Patients were more likely to undergo nephrectomy if they were younger, had lower tumor stage or N0 disease, were privately insured, or treated at an academic center. After adjusting for available covariates, patients who underwent nephrectomy had a lower risk of death (HR 0.45; 95% CI 0.40–0.50; P < 0.001). It is interesting to note that they also report on the timing of surgery for 4223 patients (88.4% had nephrectomy before and 11.6% after targeted therapy): the 1-, 2-, and 3-year OS rates were lower for those who had nephrectomy before targeted therapy (61.2%, 37.8%, and 26.6%) versus those who had nephrectomy after targeted therapy (73.3%, 48.1%, and 35.3%) (P < 0.01).Heng (29) reviewed retrospective data from 1658 patients from nine countries with synchronous mRCC treated with targeted therapies, comparing results for those who underwent nephrectomy (982) versus those who did not (676). There were significant imbalances in patient characteristics—those undergoing nephrectomy had better performance status and better International mRCC Database Consortium (IMDC) prognostic profiles. After adjustments for IMDC scores, HR for death was 0.60 (95% CI 0.52–0.69; P < 0.0001) and median OS was 20.6 versus 9.6 months benefiting the nephrectomy group. Conti et al. (17) reported survival outcomes for mRCC CN between 1993 and 2010 in the SEER database. Nephrectomy was performed in 34% (6915 out of 20,104) of the patients. Neither PS nor several other known mRCC prognostic factors were available and thus they were not considered for statistical matching in this study. Age, tumor size, sex, ethnicity, region, and other factors differed between groups, and these were adjusted for in their analysis. Relative mortality HR was 0.43 (95% CI 0.42–0.46, P < 0.05) favoring the CN arm. Abern (30) also reviewed the SEER database between 2005 and 2009, where 2629 out of 7143 patients (37%) underwent CN. Once again, patient characteristics differed significantly—patients undergoing CN were younger and more likely to be white, male, and married. In this series, patients that underwent CN had improved 1-year survival (61% vs. 22%), and surgery was associated with improved overall survival (HR = 0.40, 95% CI 0.37–0.43) on multivariable analysis.

**Table 1:** Large (>1.000 patients) retrospective cohort studies evaluating the effect of cytoreductive nephrectomy on overall survival in mRCC.

Reference (year)	Participants (*n*)	Treatment arms (*n*)	Median age (years)	Poor PS (KPS > 80 or ECOG ≥ 2)	Poor patient risk category (IMDC or MSKCC)	Clinical stage T1	mOS (months)
Hanna et al. (2016) (28)	15,390	CN (5374)	60	NR	NR	29.5%	32.5
		No CN (10,016)	64	NR	NR	15.3%	14.9
Heng et al. (2014) (29)	1658	CN (982)	60	19%	28%	NR	20.6
		No CN (676)	59	42%	54%	NR	9.6
Conti et al. (2014) (17)	20,104	CN (6915)	61	NR	NR	NR	15
		No CN (13,189)	68	NR	NR	NR	4
Abern et al. (2014) (30)	7143	CN (2629)	61	NR	NR	15%	NR
		No CN (4514)	68	NR	NR	19%	NR

Statistically significant differences in bold.

NR, not reported; CN, cytoreductive nephrectomy; PS, performance status; MSKCC, Memorial Sloan Kettering Cancer Center; mOS, median overall survival; KPS, Karnofsky Performance Scale; ECOG, Eastern Cooperative Oncology Group; IMDC, International mRCC Database Consortium.

## Cytoreductive Nephrectomy in the Targeted Therapies Era: Prospective Data

The SURTIME trial (EORTC 30073) (31) was designed to compare two different strategies, both of which included nephrectomy: the traditional approach of upfront CN followed by targeted therapy (sunitinib) versus upfront sunitinib with delayed CN after three cycles. So even though the SURTIME trial does not intend to answer the question whether we still need CN in mRCC, it sheds light on the best timing to perform it. Arms were well balanced between immediate and deferred surgery; median age was 60 and 58 years, respectively; MSKCC intermediate risk was reported for 86% versus 87.7% of patients; WHO PS 0 accounted for 72% versus 63.3% of patients; and mean primary tumor size was 93.1 mm versus 96.8 mm. Trial results showed equivalent progression-free rate (PFR) at 28 weeks: 42.0% (95% CI 28.2–56.8) and 42.9% (28.8–57.8) in the immediate and deferred arms, respectively (P > 0.99). The OS analysis favored deferred versus immediate CN with HR 0.57 (95% CI 0.34–0.95, P = 0.032) and a twofold median OS advantage: 32.4 (95% CI 14.5–65.3) and 15.1 months (95% CI 9.3–29.5), respectively. Surgical complications occurred less frequently in the deferred CN arm (27.5%) compared to the upfront CN arm (43.5%).

The Clinical Trial to Assess the Importance of Nephrectomy (CARMENA) (32) is by far the largest trial in this setting, and the only one to include TKIs. It is a prospective, randomized, open-label, non-inferiority trial where 450 mRCC patients were randomized in a 1:1 ratio to undergo nephrectomy followed by sunitinib or to be administered sunitinib alone. Eligible patients had to be suitable candidates for nephrectomy— >18 years of age, Eastern Cooperative Oncology Group (ECOG) performance status score 0 or 1, absent or treated brain metastases, and acceptable organ function. Randomization was stratified according to MSKCC prognostic groups, identified as intermediate (55.6%) and poor risk (44.6%) in the nephrectomy plus sunitinib arm versus intermediate (58.5%) and poor risk (41.5%) in the sunitinib alone arm. Median primary tumor size was 86 mm for the sunitinib arm and 88 mm for the surgery arm. Median number of metastatic sites was 2 (range 1–5) for both arms. Sunitinib 50 mg was administered in a 4 weeks on/2 weeks off schedule. Median OS, which was the primary endpoint of the study, was 18.4 months for sunitinib alone versus 13.9 months in the sunitinib plus nephrectomy group; HR for death was 0.89 (95% CI 0.71–1.10; upper boundary of the 95% confidence interval for noninferiority ≤ 1.20). In an exploratory analysis, the MSKCC intermediate risk subgroup median OS was 19.0 months with CN plus sunitinib and 23.4 months with sunitinib alone (HR = 0.92; 95% CI 0.60–1.24); in the case of MSKCC poor-risk patients, it was 10.2 and 13.3 months, respectively (HR = 0.86; 95% CI 0.62–1.17). No significant differences in ORR or progression-free survival (PFS) were observed. Median duration of sunitinib treatment was longer in the sunitinib-alone arm (8.5 vs 6.7 months, P = 0.04), and dose reductions were equally frequent in both arms (30.6% vs 30.5%, respectively). An overview of these prospective trials is summarized in Table 2.

**Table 2:** Prospective trials comparing nephrectomy followed by systemic therapy versus systemic therapy alone in mRCC.

Reference (year)	Participants (*n*)	Treatment arms	Median age (years)	ECOG PS 0–1 (%)	Patient risk (MSKCC)	ORR (%)	mOS (months)
Méjean et al. (2018) (32)	450	CN + Sunitinib	63	0 (57.5%) 1 (42.5%)	Inter (55.6%)	27.4%	13.9
		Sunitinib	62	0 (54.5%) 1 (45.5%)	Poor (44.4%)	29.1%	18.4
Flanigan et al. (2001) (4)	120	CN + IFNa	59	0 (48%) 1 (52%)	Not available	3.3%	11.1
		IFNa	59	0 (40%) 1 (60%)	Not available	3.6%	8.1
Mickisch et al. (2001) (5)	85	CN + IFNa	61	0 (55%) 1 (45%)	Not available	19%	17
		IFNa	56	0 (42%) 1 (58%)	Not available	12%	7

NR, not reported; CN, cytoreductive nephrectomy; PS, performance status; IFNa, interferon alpha; MSKCC, Memorial Sloan Kettering Cancer Center; ORR, objective response rate; mOS, median overall survival; ECOG, Eastern Cooperative Oncology Group.

## Benefit Discrepancy between Retrospective and Prospective Data in the Post-Interferon Era

The available retrospective studies we discussed previously consistently show an overall survival advantage for the combination approach with upfront CN followed by targeted therapy, which contrasts with the prospective CARMENA trial showing that targeted therapy alone is non-inferior for OS compared to the combination of CN followed by targeted therapy, and also the SURTIME trial which shows that delayed nephrectomy has similar PFR and a possible OS advantage compared to immediate CN. This could be explained by several factors.

Retrospective trials in this setting are prone to patient selection bias, as all the trials we discussed show that patients selected for surgery were usually younger, had better performance status and had better prognostic scores. All publications tried to address this by adjusting for prognostic factors; however, these adjustments were limited to what was collected and available in the source database, so this bias was not fully accounted for in any of the reviewed studies. Another significant difference is that both prospective trials included only patients with good performance status (PS 0-1), as most patients in PS 2 or more would not be candidates for surgery in the real-world setting; retrospective trials did not have the same criteria. There is also the possibility of publication bias, with negative results for nephrectomy series less likely to be published in peer-reviewed journals. It is also important to note that three out the four discussed retrospective series analyzed the same SEER registry (although with differing time frames and criteria), and thus, similar results were to be expected.

Regarding prospective trials, SURTIME did not reach its initial accrual objective, and after 5.7 years, it included just 99 patients from 19 institutions; given the poor accrual, it was decided to report the PFR at week 28 as the primary endpoint, which required 98 patients, instead of the original median PFS, which required 380 events to detect a 3-month increase. That means, the study, although positive for its PFR primary objective, was underpowered for the OS analysis, and the advantage seen in the delayed nephrectomy arm should be taken as an exploratory analysis.

The CARMENA trial also accrued slowly, taking 8 years to complete. It included a high percentage of poor-risk patients (44 and 41% in the CN plus sunitinib and sunitinib-alone arms, respectively); retrospective data have suggested that poor-risk patients derive limited or no benefit from CN,^34^ but given these were good PS patients with high tumor burden, it is still clinically relevant. Patients in the nephrectomy-sunitinib arm also had a higher rate of T3/T4 disease than those in the sunitinib arm: 70.1% versus 51%, although median primary tumor size was similar. It is also important to note that a significant number of patients (17%) in the sunitinib-only arm underwent nephrectomy because of acute symptoms or incomplete response.

## Future Landscape

Both prospective trials with targeted therapies that we have reviewed used sunitinib as upfront systemic therapy, which mirror current clinical practice as anti-VEGF TKIs have been the mainstay in the first-line setting the last 10 years (33). This scenario is changing, moving towards immunotherapy-based combinations. In the pivotal CheckMate 214 trial (14), nivolumab plus ipilimumab showed superior OS compared to sunitinib in the first–line setting; this combination was approved by the FDA in April 2018 for IMDC intermediate- and poor-risk patients. In the phase III IMmotion 151 trial (34), atezolizumab plus bevacizumab showed superior PFS compared to sunitinib, with OS data still immature at the first interim analysis. Another combination showing great activity on earlier phases is pembrolizumab plus axitinib, as reported by Atkins et al. (35) in open-label phase Ib trial in 52 mRCC patients: ORR was 73% and median PFS was 20.9 months. As these therapies eventually move into the clinical practice, we will need to reevaluate the role of CN.

## Conclusions

The advances in management of mRCC since 2005 have been rapid, and standards of care are changing, with several new effective treatment options available. In this scenario, and given the new evidence available, CN still plays an important role in the management of mRCC, despite the morbidity and mortality associated with the procedure. We believe most patients with low metastatic burden and good performance status should be offered CN, while patients with poor PS and advanced age will probably not benefit from surgery. In that advanced age scenario, systemic therapy alone could be an option, especially if tumor burden is high, whether if it´s low, observation would be our first alternative. As for patients with good PS and high tumor burden, the CARMENA trial provides the best currently available evidence, and we should act accordingly: upfront CN should not be considered standard of care, but careful consideration of not only disease-specific risk scores but also surgical risk, resectability, morbidity, and the patient’s personal preference must be taken into account in the decision-making process.

The rapid advances in the field are unlikely to slow any time soon, with sunitinib being increasingly challenged by newer agents and immunotherapy-based combinations in the first-line setting. Whether CN continues to play a role in the future will need to be evaluated prospectively.

## Conflict of interest

The authors declare no potential conflicts of interest with respect to research, authorship, and/or publication of this article.
